# Supplementary material for: Physiological and Transcriptomic Responses of Growth in Neolamarckia cadamba Stimulated by Exogenous Gibberellins
Source: Int J Mol Sci. 2022 Oct 6;23(19):11842. doi: 10.3390/ijms231911842 (PMC9569647; doi:10.3390/ijms231911842)
Supplement: Supplementary file 1 [file ijms-23-11842-s001.zip › Supplementary Figure.pdf]

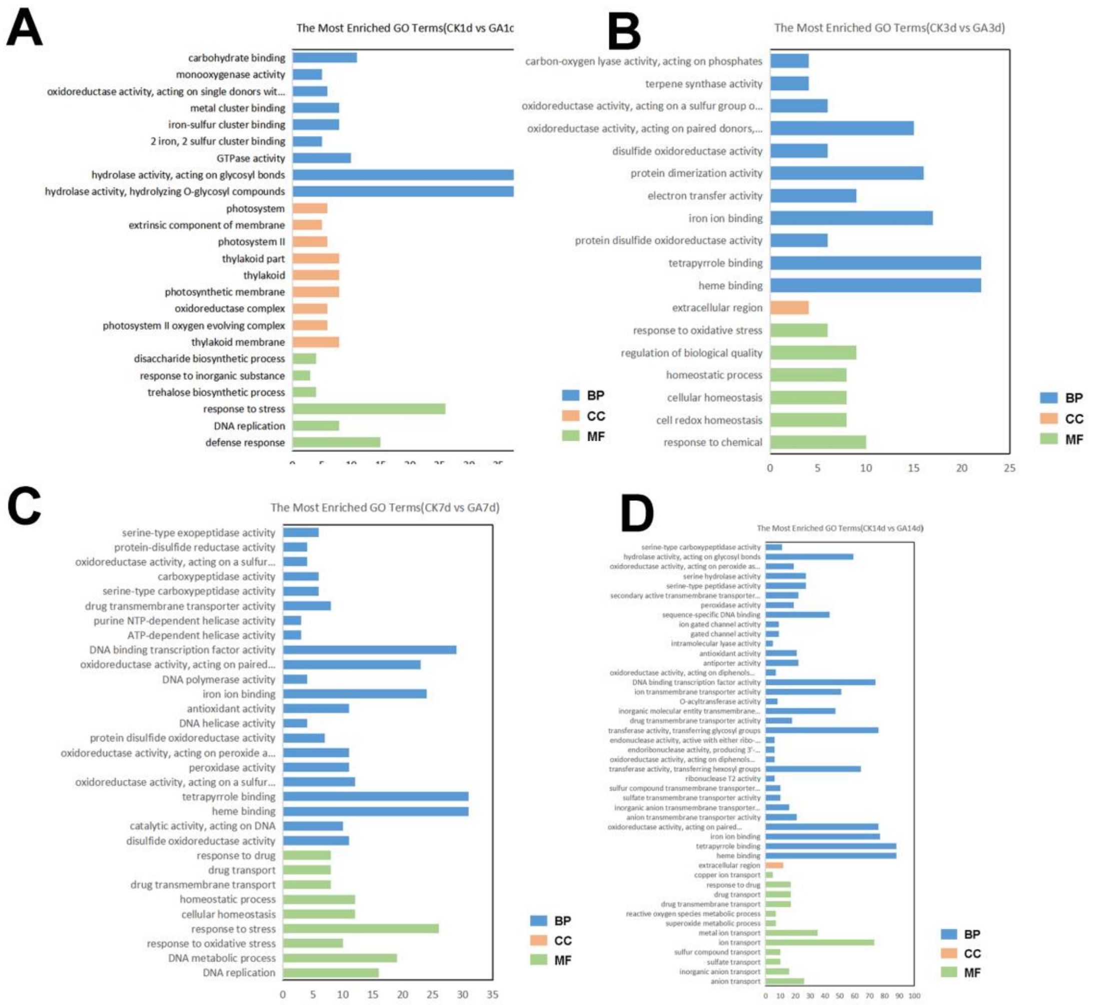

Figure S1. GO enrichment analysis of the second internode on four sets from CK groups and GA groups in *N. cadamba*. (A) GO enrichment analysis at 1 d from CK group and GA group. (B) GO enrichment analysis at 3 d from CK group and GA group. (C) GO enrichment analysis at 7 d from CK group and GA group. (D) GO enrichment analysis at 14 d from CK group and GA group. The corrected p-value ≤ 0.05 was defined as prominent enriched GO terms.

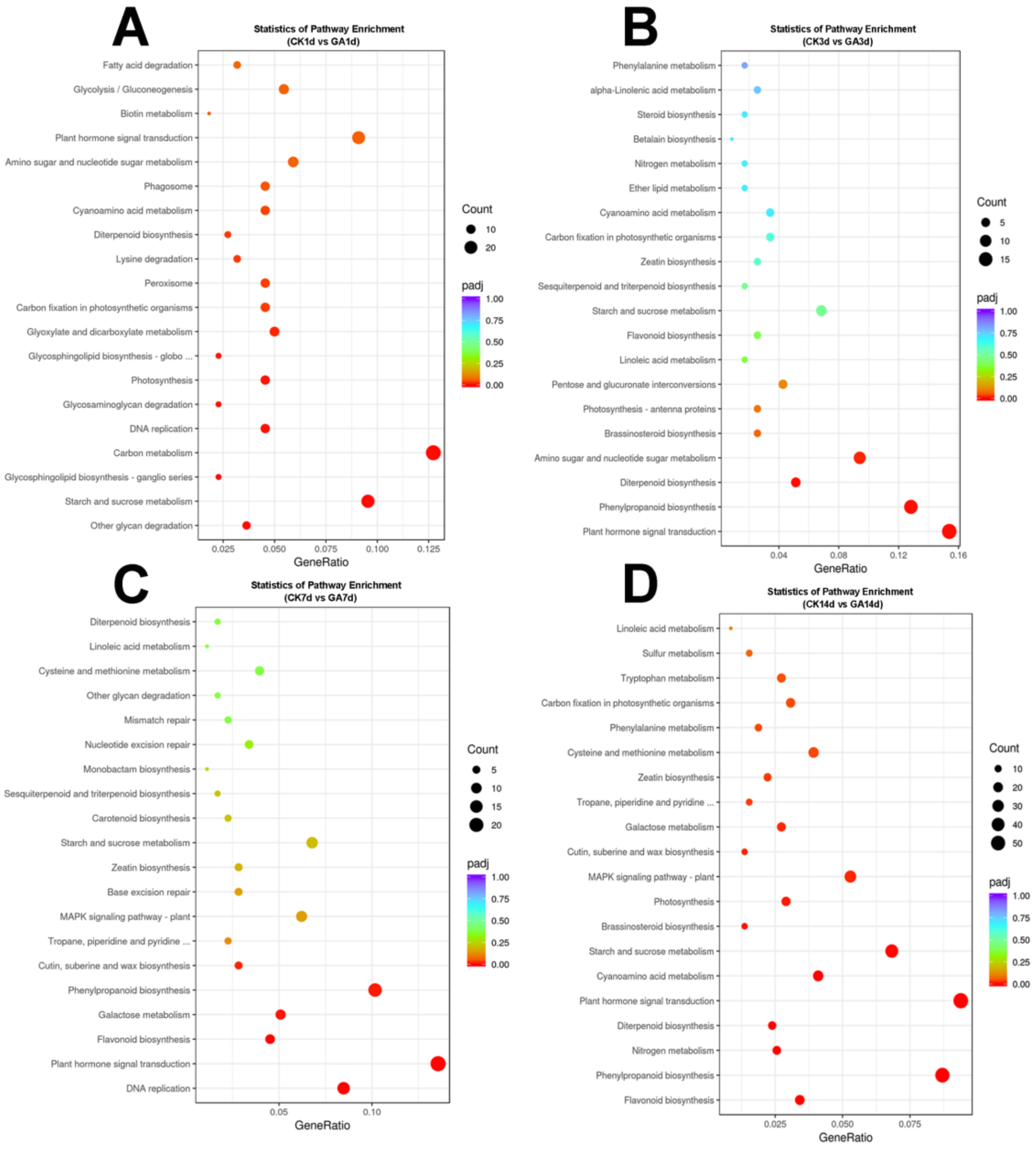

Figure S2. KEGG (Kyoto Encyclopedia of Genes and Genomes) enrichment analysis of *N. cadamba* second internode on four sets from CK group and GA group. (A) KEGG enrichment analysis at 1 d from CK group and GA group. (B) KEGG enrichment analysis at 3 d from CK group and GA group. (C) KEGG enrichment analysis at 7 d from CK group and GA group. (D) KEGG enrichment analysis at 14 d from CK group and GA group. The abscissa indicates the rich factor, the size of the dot indicates the number of differentially expressed genes in the pathway, and the color of the dot corresponds to different q value ranges.

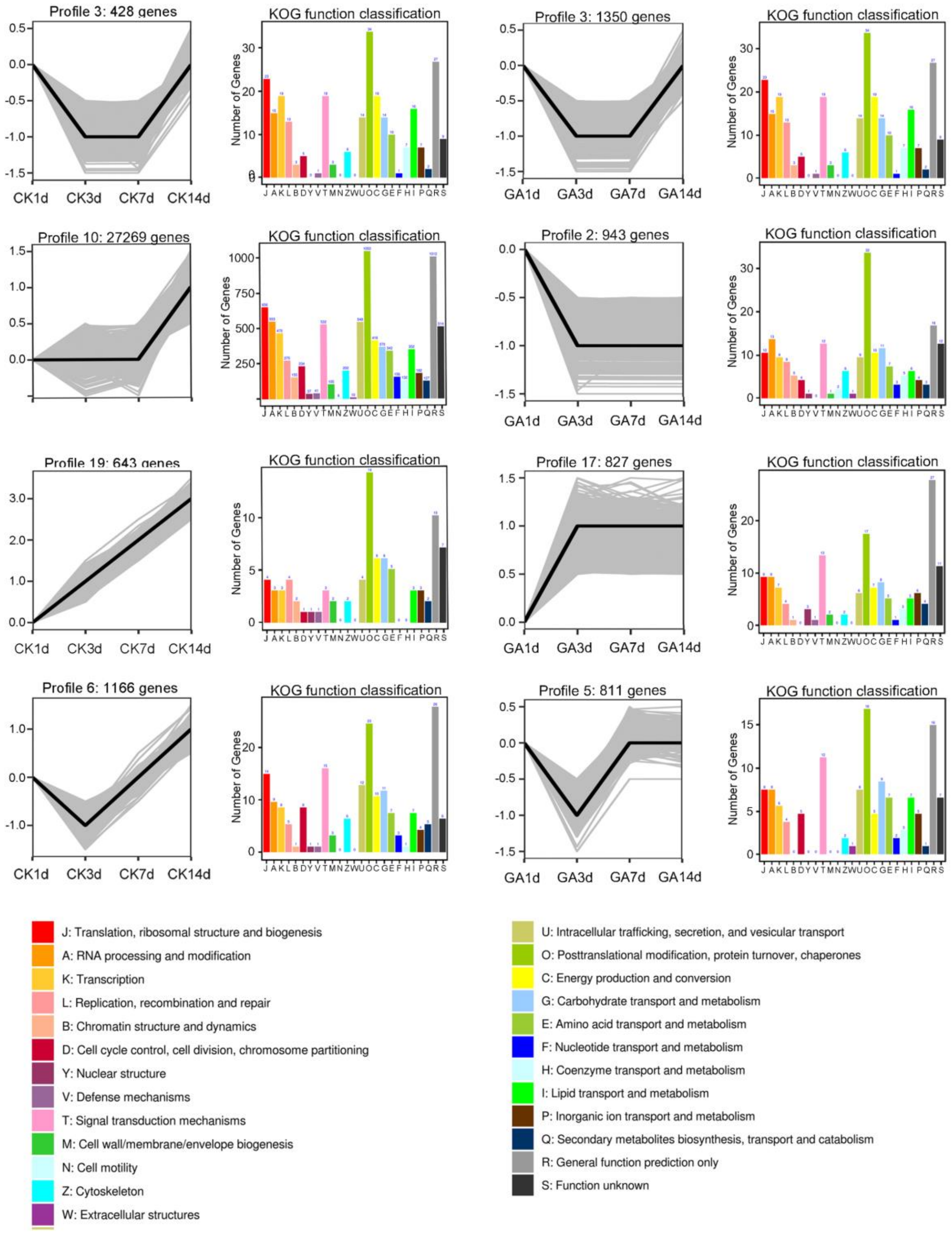

Figure S3. The expression profiles of DEGs of the second internode in *N. cadamba*. Short time-series expression miner (STEM) clustering on DEGs in 8 different patterns. The y-axis on the left side indicates the absolute value of log2 (Fold Change). The x-axis on the right side represents the number of genes in a category.

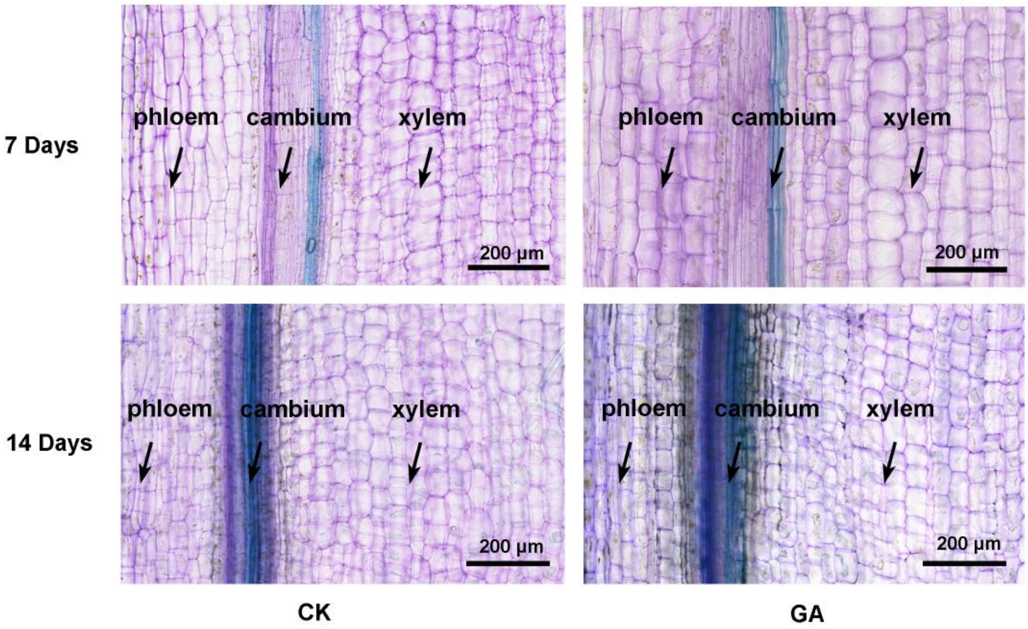

Figure S4. Longitudinal section analysis of the second internode cell at 7 d and 14 d from CK group and GA group, material stained with toluidine blue. Arrows indicate phloem, cambium and xylem. Scale bar, 200 µm.

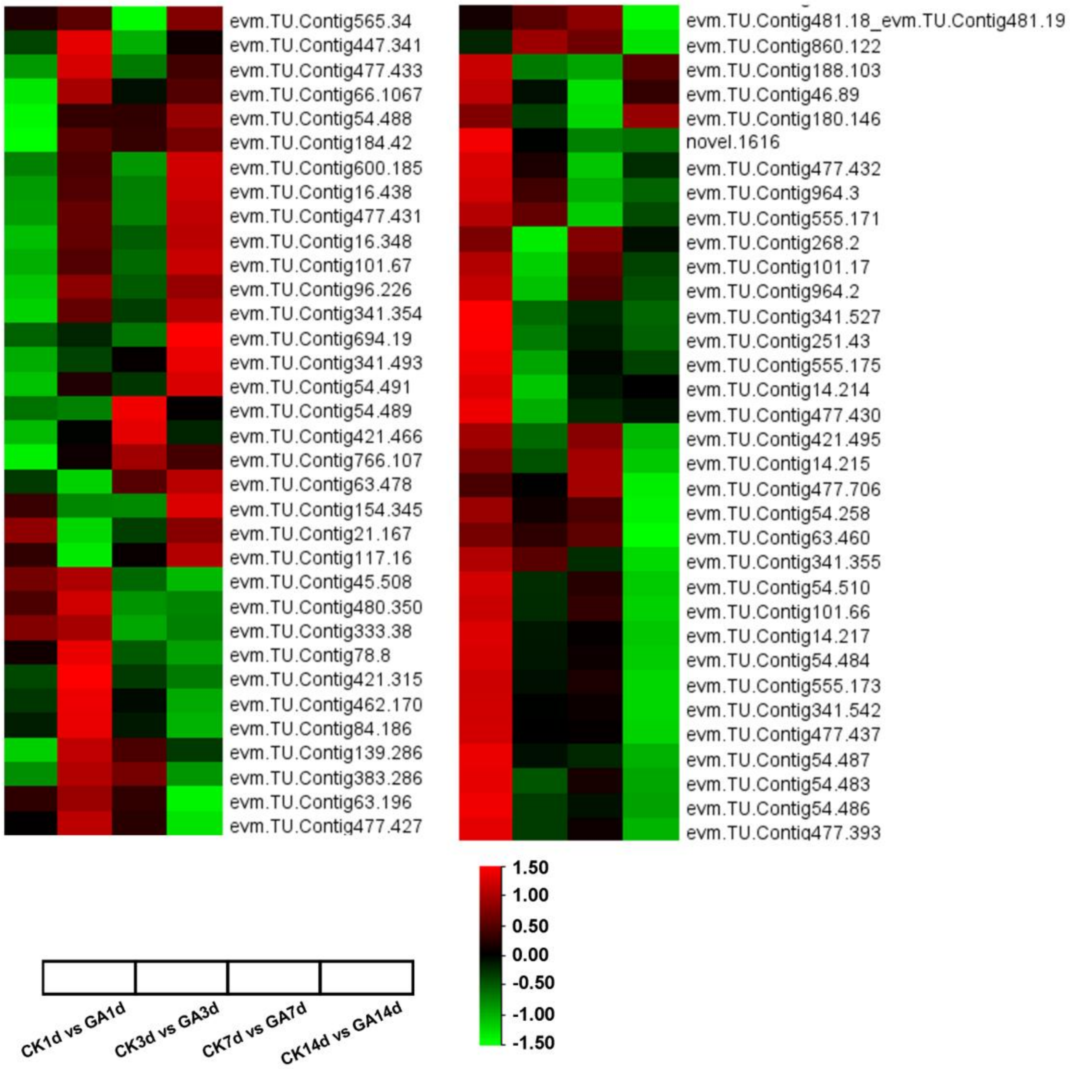

Figure S5. The analysis of DEGs associated with auxin signaling. Data shown with log2 (Fold Change) analysis. Heatmap of columns and rows represent samples and genes, respectively.

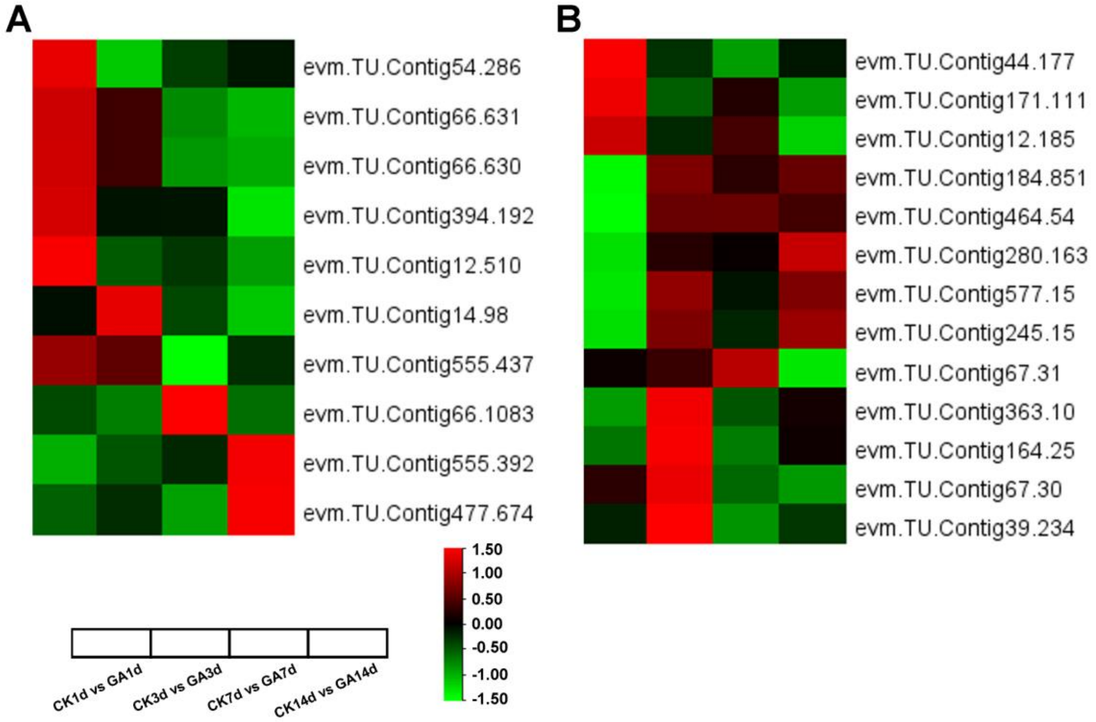

Figure S6. The analysis of DEGs associated with BR and SA signaling. (A) RNA-seq data showed the DEGs of BR signaling. (B) RNA-seq data showed the DEGs of SA signaling. Data shown with log2 (Fold Change) analysis. Heatmap of columns and rows represent samples and genes, respectively.
